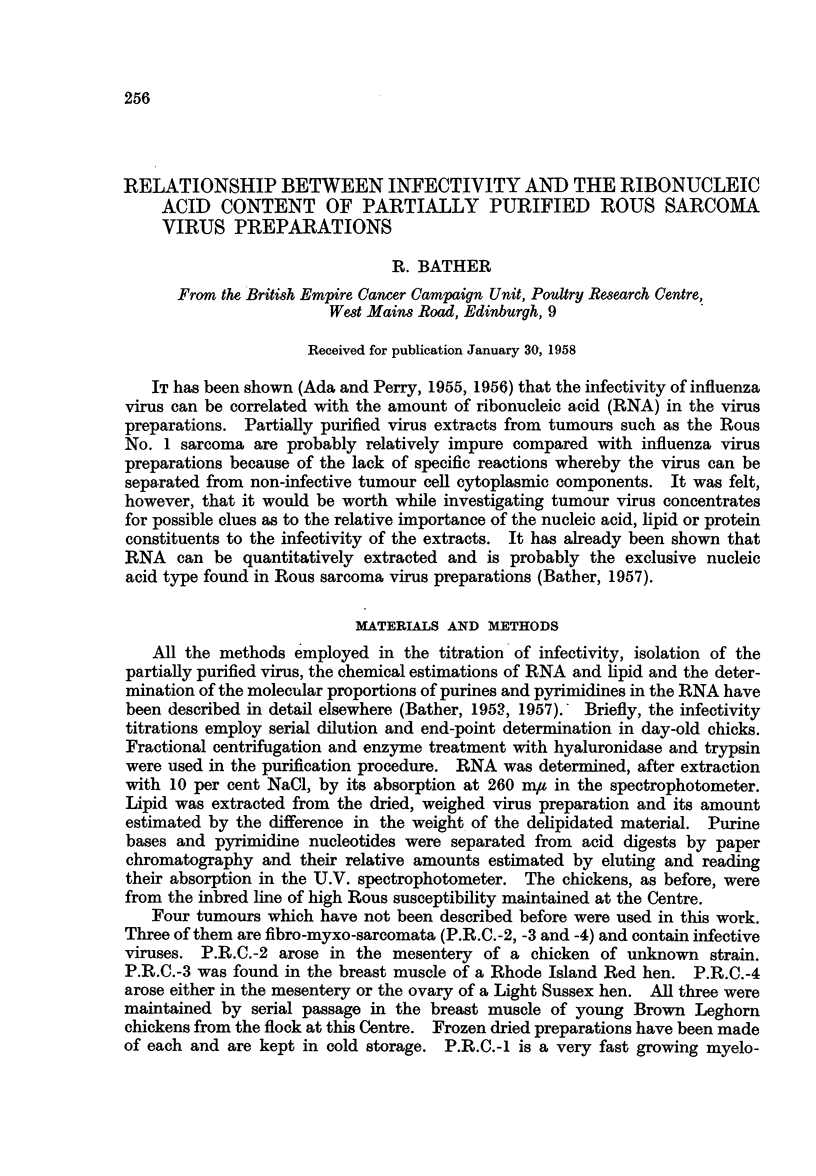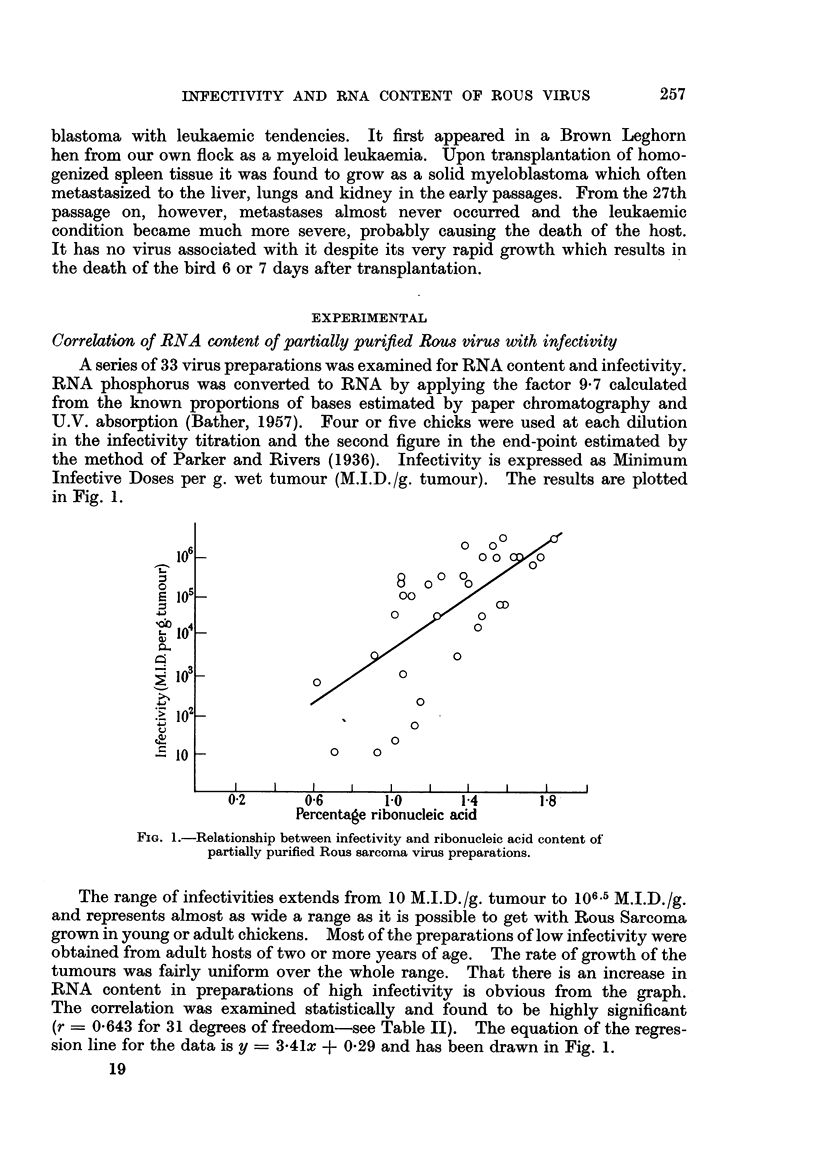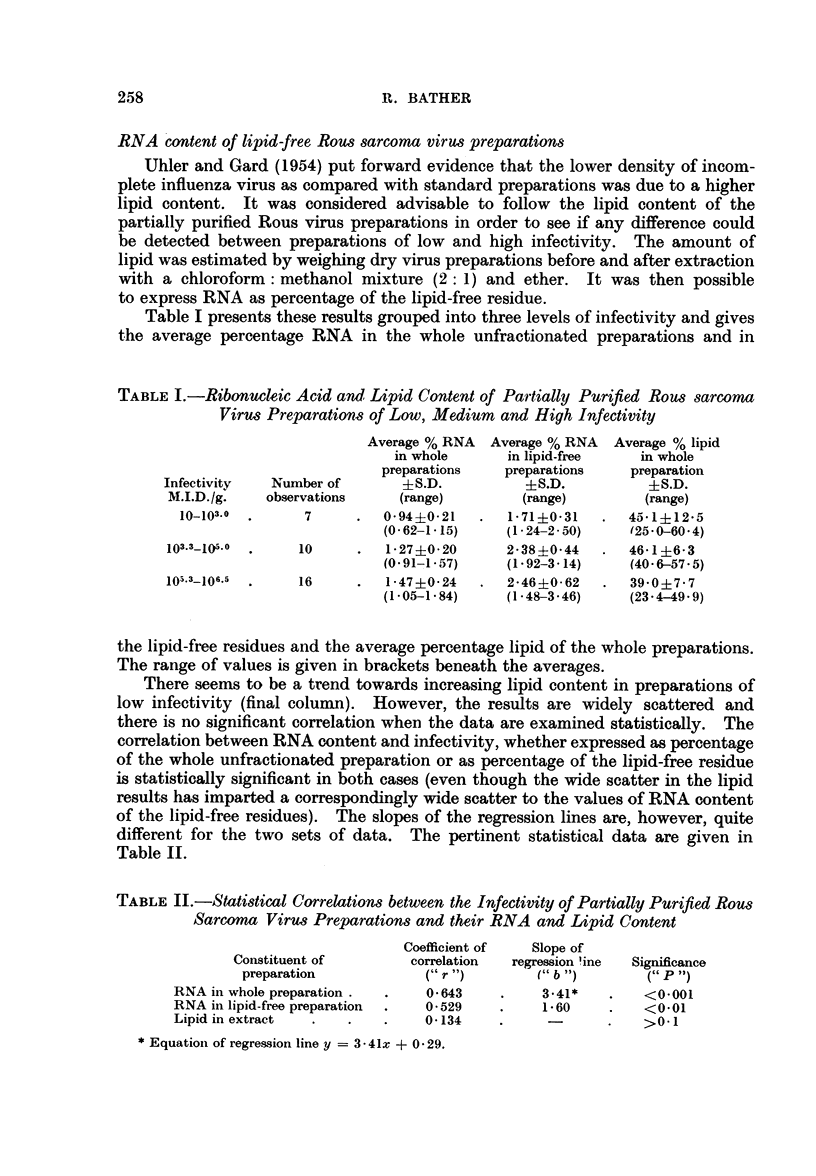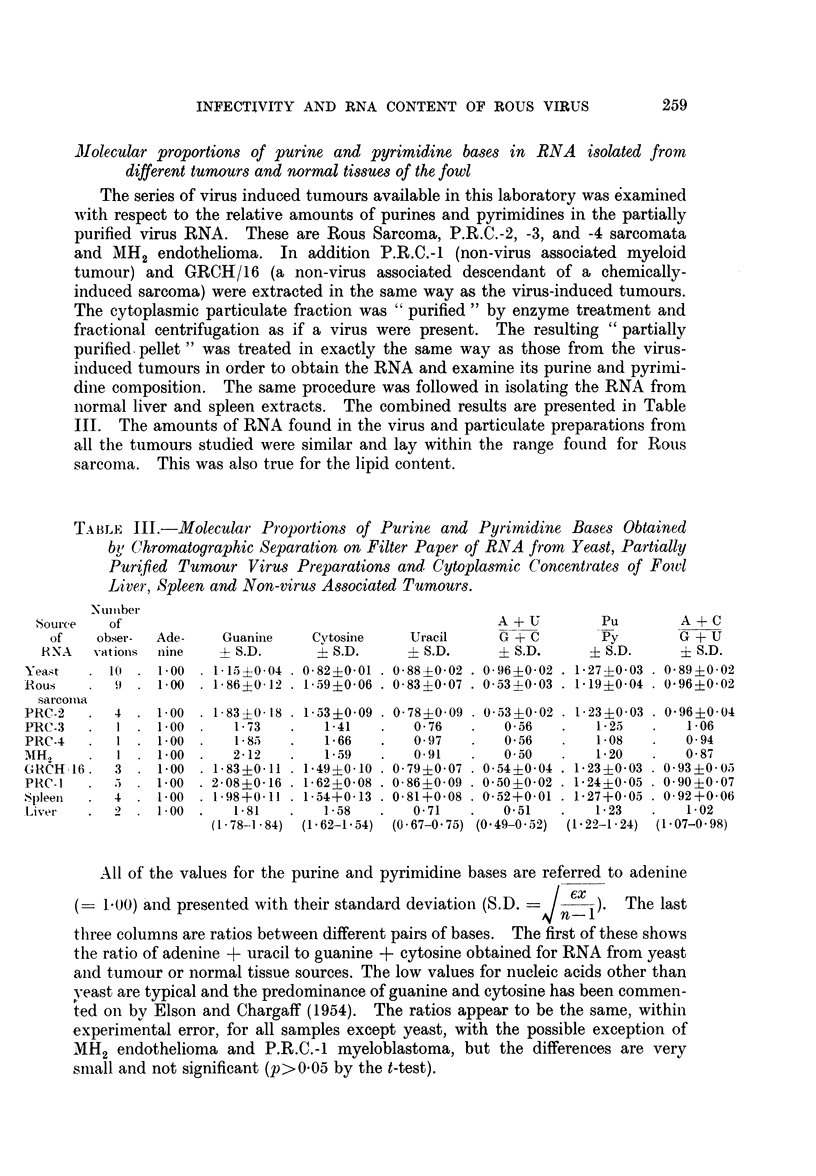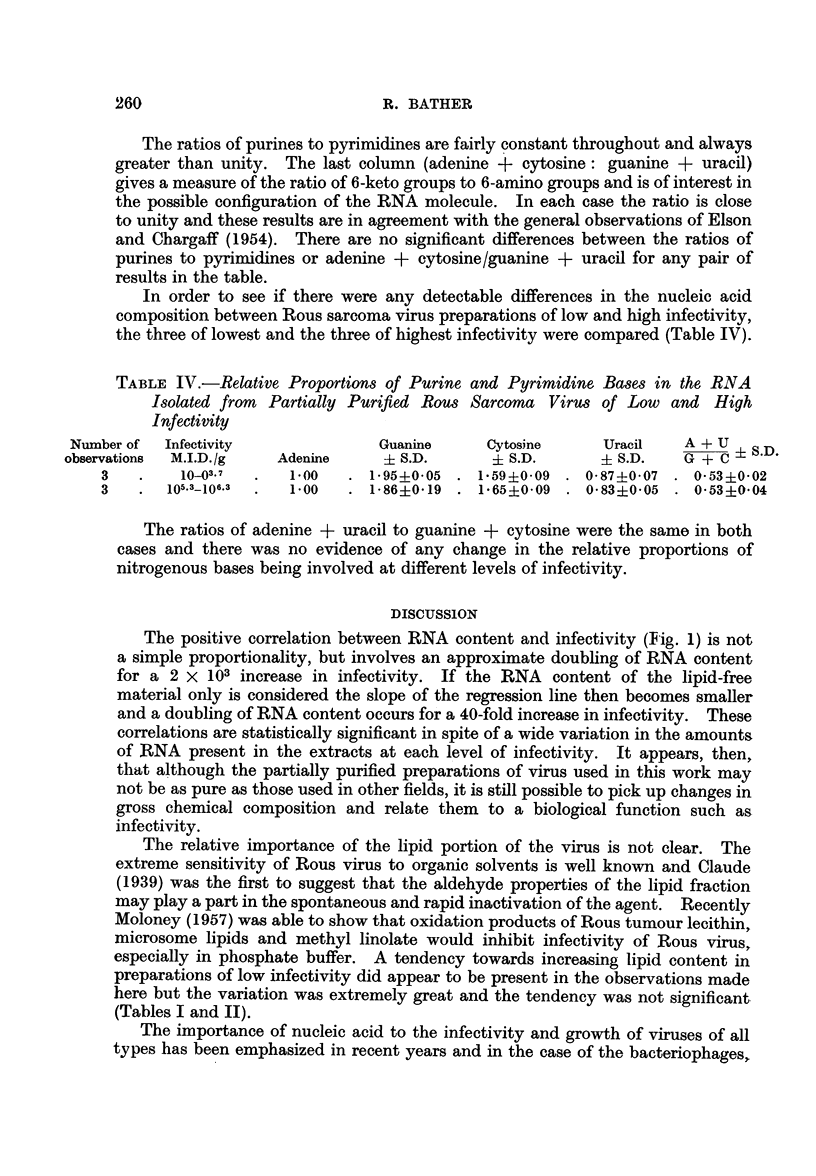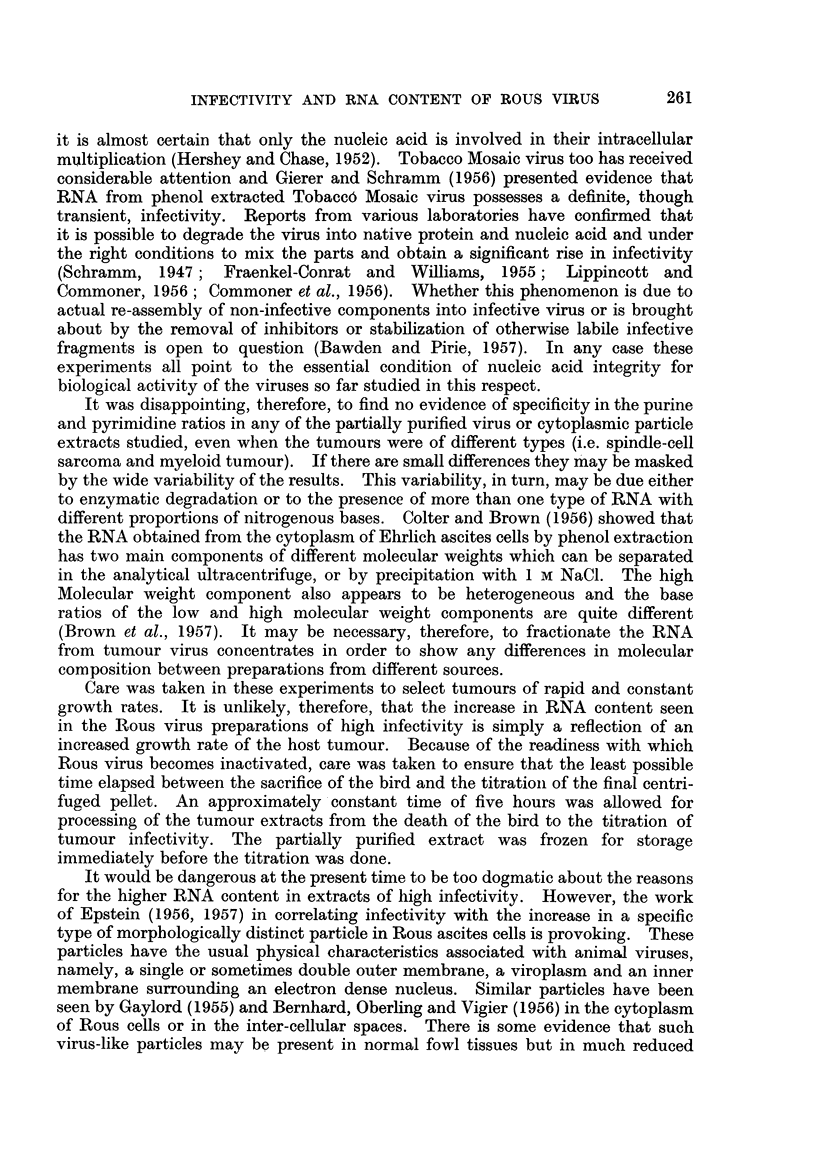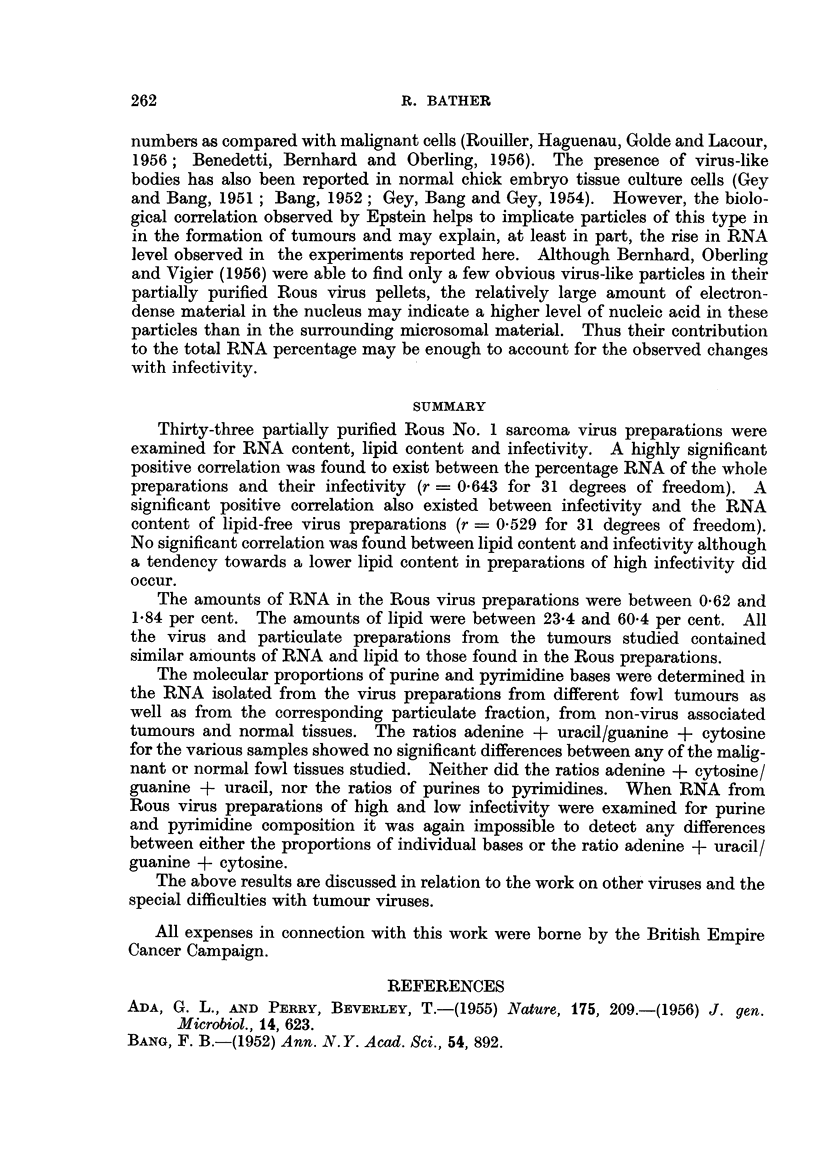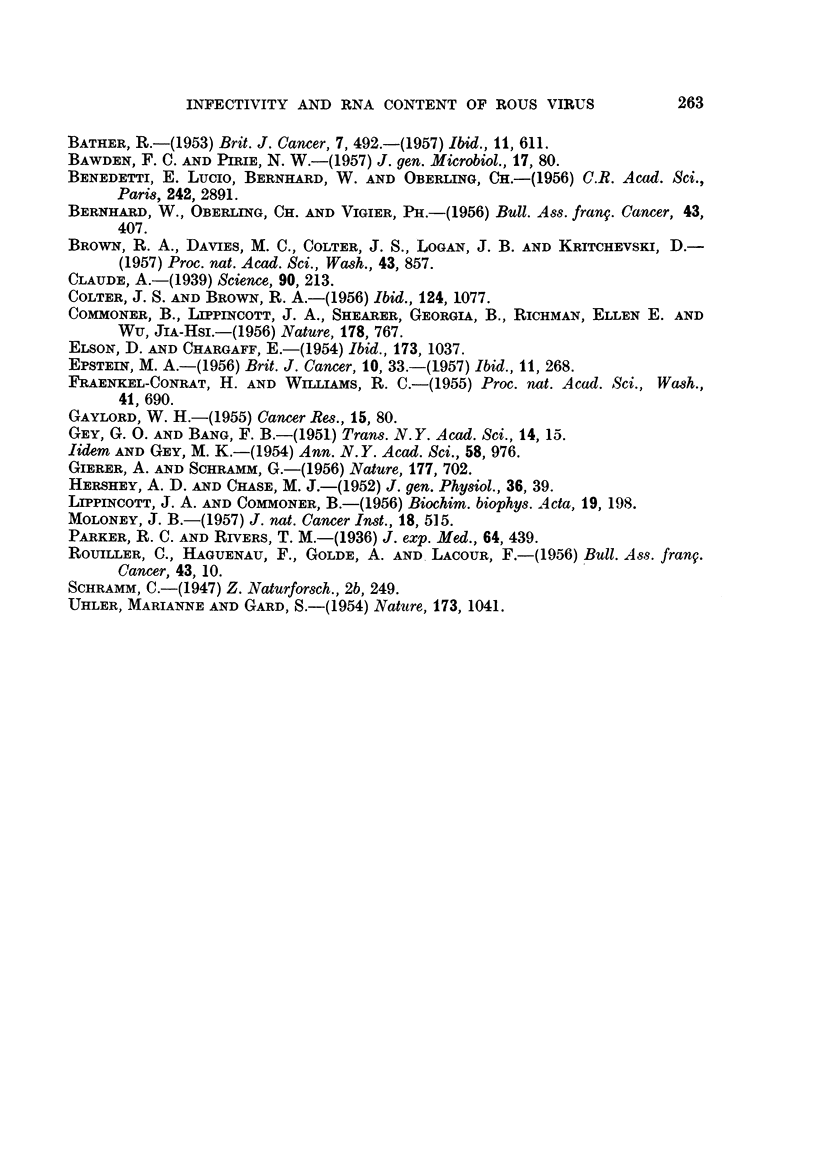# Relationship Between Infectivity and the Ribonucleic Acid Content of Partially Purified Rous Sarcoma Virus Preparations

**DOI:** 10.1038/bjc.1958.31

**Published:** 1958-06

**Authors:** R. Bather


### RELATIONSHIP BETWEEN INFECTIVITY AND THE RIBONUCLEIC ACID CONTENT OF PARTIALLY PURIFIED ROUS SARCOMA VIRUS PREPARATIONS

R. BATHER

*From the British Empire Cancer Campaign Unit, Poultry Research Centre, West Mains Road, Edinburgh, 9*

Received for publication January 30, 1958

It has been shown (Ada and Perry, 1955, 1956) that the infectivity of influenza virus can be correlated with the amount of ribonucleic acid (RNA) in the virus preparations. Partially purified virus extracts from tumours such as the Rous No. 1 sarcoma are probably relatively impure compared with influenza virus preparations because of the lack of specific reactions whereby the virus can be separated from non-infective tumour cell cytoplasmic components. It was felt, however, that it would be worth while investigating tumour virus concentrates for possible clues as to the relative importance of the nucleic acid, lipid or protein constituents to the infectivity of the extracts. It has already been shown that RNA can be quantitatively extracted and is probably the exclusive nucleic acid type found in Rous sarcoma virus preparations (Bather, 1957).

### MATERIALS AND METHODS

All the methods employed in the titration of infectivity, isolation of the partially purified virus, the chemical estimations of RNA and lipid and the determination of the molecular proportions of purines and pyrimidines in the RNA have been described in detail elsewhere (Bather, 1953, 1957). Briefly, the infectivity titrations employ serial dilution and end-point determination in day-old chicks. Fractional centrifugation and enzyme treatment with hyaluronidase and trypsin were used in the purification procedure. RNA was determined, after extraction with 10 per cent NaCl, by its absorption at 260 m$\mu$ in the spectrophotometer. Lipid was extracted from the dried, weighed virus preparation and its amount estimated by the difference in the weight of the delipidated material. Purine bases and pyrimidine nucleotides were separated from acid digests by paper chromatography and their relative amounts estimated by eluting and reading their absorption in the U.V. spectrophotometer. The chickens, as before, were from the inbred line of high Rous susceptibility maintained at the Centre.

Four tumours which have not been described before were used in this work. Three of them are fibro-myxo-sarcomata (P.R.C.-2, -3 and -4) and contain infective viruses. P.R.C.-2 arose in the mesentery of a chicken of unknown strain. P.R.C.-3 was found in the breast muscle of a Rhode Island Red hen. P.R.C.-4 arose either in the mesentery or the ovary of a Light Sussex hen. All three were maintained by serial passage in the breast muscle of young Brown Leghorn chickens from the flock at this Centre. Frozen dried preparations have been made of each and are kept in cold storage. P.R.C.-1 is a very fast growing myeloblastoma with leukaemic tendencies. It first appeared in a Brown Leghorn hen from our own flock as a myeloid leukaemia. Upon transplantation of homogenized spleen tissue it was found to grow as a solid myeloblastoma which often metastasized to the liver, lungs and kidney in the early passages. From the 27th passage on, however, metastases almost never occurred and the leukaemic condition became much more severe, probably causing the death of the host. It has no virus associated with it despite its very rapid growth which results in the death of the bird 6 or 7 days after transplantation.

### EXPERIMENTAL

*Correlation of RNA content of partially purified Rous virus with infectivity*

A series of 33 virus preparations was examined for RNA content and infectivity. RNA phosphorus was converted to RNA by applying the factor 9·7 calculated from the known proportions of bases estimated by paper chromatography and U.V. absorption (Bather, 1957). Four or five chicks were used at each dilution in the infectivity titration and the second figure in the end-point estimated by the method of Parker and Rivers (1936). Infectivity is expressed as Minimum Infective Doses per g. wet tumour (M.I.D./g. tumour). The results are plotted in Fig. 1.

FIG. 1.—Relationship between infectivity and ribonucleic acid content of partially purified Rous sarcoma virus preparations.

The range of infectivities extends from 10 M.I.D./g. tumour to $10^{6.5}$ M.I.D./g. and represents almost as wide a range as it is possible to get with Rous Sarcoma grown in young or adult chickens. Most of the preparations of low infectivity were obtained from adult hosts of two or more years of age. The rate of growth of the tumours was fairly uniform over the whole range. That there is an increase in RNA content in preparations of high infectivity is obvious from the graph. The correlation was examined statistically and found to be highly significant ($r = 0{\cdot}643$ for 31 degrees of freedom—see Table II). The equation of the regression line for the data is $y = 3{\cdot}41x + 0{\cdot}29$ and has been drawn in Fig. 1.

*RNA content of lipid-free Rous sarcoma virus preparations*

Uhler and Gard (1954) put forward evidence that the lower density of incomplete influenza virus as compared with standard preparations was due to a higher lipid content. It was considered advisable to follow the lipid content of the partially purified Rous virus preparations in order to see if any difference could be detected between preparations of low and high infectivity. The amount of lipid was estimated by weighing dry virus preparations before and after extraction with a chloroform : methanol mixture (2 : 1) and ether. It was then possible to express RNA as percentage of the lipid-free residue.

Table I presents these results grouped into three levels of infectivity and gives the average percentage RNA in the whole unfractionated preparations and in

TABLE I.—*Ribonucleic Acid and Lipid Content of Partially Purified Rous sarcoma Virus Preparations of Low, Medium and High Infectivity*

| Infectivity M.I.D./g. | Number of observations | Average % RNA in whole preparations ±S.D. (range) | Average % RNA in lipid-free preparations ±S.D. (range) | Average % lipid in whole preparation ±S.D. (range) |
|---|---|---|---|---|
| $10–10^{3.0}$ | 7 | 0·94±0·21 (0·62–1·15) | 1·71±0·31 (1·24–2·50) | 45·1±12·5 (25·0–60·4) |
| $10^{3.3}–10^{5.0}$ | 10 | 1·27±0·20 (0·91–1·57) | 2·38±0·44 (1·92–3·14) | 46·1±6·3 (40·6–57·5) |
| $10^{5.3}–10^{6.5}$ | 16 | 1·47±0·24 (1·05–1·84) | 2·46±0·62 (1·48–3·46) | 39·0±7·7 (23·4–49·9) |

the lipid-free residues and the average percentage lipid of the whole preparations. The range of values is given in brackets beneath the averages.

There seems to be a trend towards increasing lipid content in preparations of low infectivity (final column). However, the results are widely scattered and there is no significant correlation when the data are examined statistically. The correlation between RNA content and infectivity, whether expressed as percentage of the whole unfractionated preparation or as percentage of the lipid-free residue is statistically significant in both cases (even though the wide scatter in the lipid results has imparted a correspondingly wide scatter to the values of RNA content of the lipid-free residues). The slopes of the regression lines are, however, quite different for the two sets of data. The pertinent statistical data are given in Table II.

TABLE II.—*Statistical Correlations between the Infectivity of Partially Purified Rous Sarcoma Virus Preparations and their RNA and Lipid Content*

| Constituent of preparation | Coefficient of correlation (" $r$ ") | Slope of regression line (" $b$ ") | Significance (" $P$ ") |
|---|---|---|---|
| RNA in whole preparation | 0·643 | 3·41* | $<0·001$ |
| RNA in lipid-free preparation | 0·529 | 1·60 | $<0·01$ |
| Lipid in extract | 0·134 | — | $>0·1$ |

* Equation of regression line $y = 3·41x + 0·29$.

*Molecular proportions of purine and pyrimidine bases in RNA isolated from different tumours and normal tissues of the fowl*

The series of virus induced tumours available in this laboratory was examined with respect to the relative amounts of purines and pyrimidines in the partially purified virus RNA. These are Rous Sarcoma, P.R.C.-2, -3, and -4 sarcomata and $MH_2$ endothelioma. In addition P.R.C.-1 (non-virus associated myeloid tumour) and GRCH/16 (a non-virus associated descendant of a chemically-induced sarcoma) were extracted in the same way as the virus-induced tumours. The cytoplasmic particulate fraction was " purified " by enzyme treatment and fractional centrifugation as if a virus were present. The resulting " partially purified pellet " was treated in exactly the same way as those from the virus-induced tumours in order to obtain the RNA and examine its purine and pyrimidine composition. The same procedure was followed in isolating the RNA from normal liver and spleen extracts. The combined results are presented in Table III. The amounts of RNA found in the virus and particulate preparations from all the tumours studied were similar and lay within the range found for Rous sarcoma. This was also true for the lipid content.

TABLE III.—*Molecular Proportions of Purine and Pyrimidine Bases Obtained by Chromatographic Separation on Filter Paper of RNA from Yeast, Partially Purified Tumour Virus Preparations and Cytoplasmic Concentrates of Fowl Liver, Spleen and Non-virus Associated Tumours.*

| Source of RNA | Number of obser- vations | Ade- nine | Guanine ± S.D. | Cytosine ± S.D. | Uracil ± S.D. | $\frac{A+U}{G+C}$ ± S.D. | $\frac{Pu}{Py}$ ± S.D. | $\frac{A+C}{G+U}$ ± S.D. |
|---|---|---|---|---|---|---|---|---|
| Yeast | 10 | 1·00 | 1·15±0·04 | 0·82±0·01 | 0·88±0·02 | 0·96±0·02 | 1·27±0·03 | 0·89±0·02 |
| Rous sarcoma | 9 | 1·00 | 1·86±0·12 | 1·59±0·06 | 0·83±0·07 | 0·53±0·03 | 1·19±0·04 | 0·96±0·02 |
| PRC-2 | 4 | 1·00 | 1·83±0·18 | 1·53±0·09 | 0·78±0·09 | 0·53±0·02 | 1·23±0·03 | 0·96±0·04 |
| PRC-3 | 1 | 1·00 | 1·73 | 1·41 | 0·76 | 0·56 | 1·25 | 1·06 |
| PRC-4 | 1 | 1·00 | 1·85 | 1·66 | 0·97 | 0·56 | 1·08 | 0·94 |
| $MH_2$ | 1 | 1·00 | 2·12 | 1·59 | 0·91 | 0·50 | 1·20 | 0·87 |
| GRCH/16 | 3 | 1·00 | 1·83±0·11 | 1·49±0·10 | 0·79±0·07 | 0·54±0·04 | 1·23±0·03 | 0·93±0·05 |
| PRC-1 | 5 | 1·00 | 2·08±0·16 | 1·62±0·08 | 0·86±0·09 | 0·50±0·02 | 1·24±0·05 | 0·90±0·07 |
| Spleen | 4 | 1·00 | 1·98+0·11 | 1·54+0·13 | 0·81+0·08 | 0·52+0·01 | 1·27+0·05 | 0·92+0·06 |
| Liver | 2 | 1·00 | 1·81 (1·78–1·84) | 1·58 (1·62–1·54) | 0·71 (0·67–0·75) | 0·51 (0·49–0·52) | 1·23 (1·22–1·24) | 1·02 (1·07–0·98) |

All of the values for the purine and pyrimidine bases are referred to adenine (= 1·00) and presented with their standard deviation (S.D. $= \sqrt{\frac{\epsilon x}{n-1}}$). The last three columns are ratios between different pairs of bases. The first of these shows the ratio of adenine + uracil to guanine + cytosine obtained for RNA from yeast and tumour or normal tissue sources. The low values for nucleic acids other than yeast are typical and the predominance of guanine and cytosine has been commented on by Elson and Chargaff (1954). The ratios appear to be the same, within experimental error, for all samples except yeast, with the possible exception of $MH_2$ endothelioma and P.R.C.-1 myeloblastoma, but the differences are very small and not significant ($p > 0·05$ by the *t*-test).

The ratios of purines to pyrimidines are fairly constant throughout and always greater than unity. The last column (adenine + cytosine : guanine + uracil) gives a measure of the ratio of 6-keto groups to 6-amino groups and is of interest in the possible configuration of the RNA molecule. In each case the ratio is close to unity and these results are in agreement with the general observations of Elson and Chargaff (1954). There are no significant differences between the ratios of purines to pyrimidines or adenine + cytosine/guanine + uracil for any pair of results in the table.

In order to see if there were any detectable differences in the nucleic acid composition between Rous sarcoma virus preparations of low and high infectivity, the three of lowest and the three of highest infectivity were compared (Table IV).

TABLE IV.—*Relative Proportions of Purine and Pyrimidine Bases in the RNA Isolated from Partially Purified Rous Sarcoma Virus of Low and High Infectivity*

| Number of observations | Infectivity M.I.D./g | Adenine | Guanine ± S.D. | Cytosine ± S.D. | Uracil ± S.D. | $\frac{A + U}{G + C}$ ± S.D. |
|---|---|---|---|---|---|---|
| 3 | $10–0^{3.7}$ | 1·00 | 1·95±0·05 | 1·59±0·09 | 0·87±0·07 | 0·53±0·02 |
| 3 | $10^{5.3}–10^{6.3}$ | 1·00 | 1·86±0·19 | 1·65±0·09 | 0·83±0·05 | 0·53±0·04 |

The ratios of adenine + uracil to guanine + cytosine were the same in both cases and there was no evidence of any change in the relative proportions of nitrogenous bases being involved at different levels of infectivity.

### DISCUSSION

The positive correlation between RNA content and infectivity (Fig. 1) is not a simple proportionality, but involves an approximate doubling of RNA content for a $2 \times 10^3$ increase in infectivity. If the RNA content of the lipid-free material only is considered the slope of the regression line then becomes smaller and a doubling of RNA content occurs for a 40-fold increase in infectivity. These correlations are statistically significant in spite of a wide variation in the amounts of RNA present in the extracts at each level of infectivity. It appears, then, that although the partially purified preparations of virus used in this work may not be as pure as those used in other fields, it is still possible to pick up changes in gross chemical composition and relate them to a biological function such as infectivity.

The relative importance of the lipid portion of the virus is not clear. The extreme sensitivity of Rous virus to organic solvents is well known and Claude (1939) was the first to suggest that the aldehyde properties of the lipid fraction may play a part in the spontaneous and rapid inactivation of the agent. Recently Moloney (1957) was able to show that oxidation products of Rous tumour lecithin, microsome lipids and methyl linolate would inhibit infectivity of Rous virus, especially in phosphate buffer. A tendency towards increasing lipid content in preparations of low infectivity did appear to be present in the observations made here but the variation was extremely great and the tendency was not significant (Tables I and II).

The importance of nucleic acid to the infectivity and growth of viruses of all types has been emphasized in recent years and in the case of the bacteriophages, it is almost certain that only the nucleic acid is involved in their intracellular multiplication (Hershey and Chase, 1952). Tobacco Mosaic virus too has received considerable attention and Gierer and Schramm (1956) presented evidence that RNA from phenol extracted Tobaccó Mosaic virus possesses a definite, though transient, infectivity. Reports from various laboratories have confirmed that it is possible to degrade the virus into native protein and nucleic acid and under the right conditions to mix the parts and obtain a significant rise in infectivity (Schramm, 1947 ; Fraenkel-Conrat and Williams, 1955 ; Lippincott and Commoner, 1956 ; Commoner *et al.*, 1956). Whether this phenomenon is due to actual re-assembly of non-infective components into infective virus or is brought about by the removal of inhibitors or stabilization of otherwise labile infective fragments is open to question (Bawden and Pirie, 1957). In any case these experiments all point to the essential condition of nucleic acid integrity for biological activity of the viruses so far studied in this respect.

It was disappointing, therefore, to find no evidence of specificity in the purine and pyrimidine ratios in any of the partially purified virus or cytoplasmic particle extracts studied, even when the tumours were of different types (i.e. spindle-cell sarcoma and myeloid tumour). If there are small differences they may be masked by the wide variability of the results. This variability, in turn, may be due either to enzymatic degradation or to the presence of more than one type of RNA with different proportions of nitrogenous bases. Colter and Brown (1956) showed that the RNA obtained from the cytoplasm of Ehrlich ascites cells by phenol extraction has two main components of different molecular weights which can be separated in the analytical ultracentrifuge, or by precipitation with 1 M NaCl. The high Molecular weight component also appears to be heterogeneous and the base ratios of the low and high molecular weight components are quite different (Brown *et al.*, 1957). It may be necessary, therefore, to fractionate the RNA from tumour virus concentrates in order to show any differences in molecular composition between preparations from different sources.

Care was taken in these experiments to select tumours of rapid and constant growth rates. It is unlikely, therefore, that the increase in RNA content seen in the Rous virus preparations of high infectivity is simply a reflection of an increased growth rate of the host tumour. Because of the readiness with which Rous virus becomes inactivated, care was taken to ensure that the least possible time elapsed between the sacrifice of the bird and the titration of the final centrifuged pellet. An approximately constant time of five hours was allowed for processing of the tumour extracts from the death of the bird to the titration of tumour infectivity. The partially purified extract was frozen for storage immediately before the titration was done.

It would be dangerous at the present time to be too dogmatic about the reasons for the higher RNA content in extracts of high infectivity. However, the work of Epstein (1956, 1957) in correlating infectivity with the increase in a specific type of morphologically distinct particle in Rous ascites cells is provoking. These particles have the usual physical characteristics associated with animal viruses, namely, a single or sometimes double outer membrane, a viroplasm and an inner membrane surrounding an electron dense nucleus. Similar particles have been seen by Gaylord (1955) and Bernhard, Oberling and Vigier (1956) in the cytoplasm of Rous cells or in the inter-cellular spaces. There is some evidence that such virus-like particles may be present in normal fowl tissues but in much reduced numbers as compared with malignant cells (Rouiller, Haguenau, Golde and Lacour, 1956; Benedetti, Bernhard and Oberling, 1956). The presence of virus-like bodies has also been reported in normal chick embryo tissue culture cells (Gey and Bang, 1951; Bang, 1952; Gey, Bang and Gey, 1954). However, the biological correlation observed by Epstein helps to implicate particles of this type in in the formation of tumours and may explain, at least in part, the rise in RNA level observed in the experiments reported here. Although Bernhard, Oberling and Vigier (1956) were able to find only a few obvious virus-like particles in their partially purified Rous virus pellets, the relatively large amount of electron-dense material in the nucleus may indicate a higher level of nucleic acid in these particles than in the surrounding microsomal material. Thus their contribution to the total RNA percentage may be enough to account for the observed changes with infectivity.

### SUMMARY

Thirty-three partially purified Rous No. 1 sarcoma virus preparations were examined for RNA content, lipid content and infectivity. A highly significant positive correlation was found to exist between the percentage RNA of the whole preparations and their infectivity ($r = 0{\cdot}643$ for 31 degrees of freedom). A significant positive correlation also existed between infectivity and the RNA content of lipid-free virus preparations ($r = 0{\cdot}529$ for 31 degrees of freedom). No significant correlation was found between lipid content and infectivity although a tendency towards a lower lipid content in preparations of high infectivity did occur.

The amounts of RNA in the Rous virus preparations were between 0·62 and 1·84 per cent. The amounts of lipid were between 23·4 and 60·4 per cent. All the virus and particulate preparations from the tumours studied contained similar amounts of RNA and lipid to those found in the Rous preparations.

The molecular proportions of purine and pyrimidine bases were determined in the RNA isolated from the virus preparations from different fowl tumours as well as from the corresponding particulate fraction, from non-virus associated tumours and normal tissues. The ratios adenine + uracil/guanine + cytosine for the various samples showed no significant differences between any of the malignant or normal fowl tissues studied. Neither did the ratios adenine + cytosine/guanine + uracil, nor the ratios of purines to pyrimidines. When RNA from Rous virus preparations of high and low infectivity were examined for purine and pyrimidine composition it was again impossible to detect any differences between either the proportions of individual bases or the ratio adenine + uracil/guanine + cytosine.

The above results are discussed in relation to the work on other viruses and the special difficulties with tumour viruses.

All expenses in connection with this work were borne by the British Empire Cancer Campaign.

### REFERENCES

ADA, G. L., AND PERRY, BEVERLEY, T.—(1955) *Nature*, **175**, 209.—(1956) *J. gen. Microbiol.*, **14**, 623.
BANG, F. B.—(1952) *Ann. N.Y. Acad. Sci.*, **54**, 892.

BATHER, R.—(1953) *Brit. J. Cancer*, **7**, 492.—(1957) *Ibid.*, **11**, 611.
BAWDEN, F. C. AND PIRIE, N. W.—(1957) *J. gen. Microbiol.*, **17**, 80.
BENEDETTI, E. LUCIO, BERNHARD, W. AND OBERLING, CH.—(1956) *C.R. Acad. Sci., Paris*, **242**, 2891.
BERNHARD, W., OBERLING, CH. AND VIGIER, PH.—(1956) *Bull. Ass. franç. Cancer*, **43**, 407.
BROWN, R. A., DAVIES, M. C., COLTER, J. S., LOGAN, J. B. AND KRITCHEVSKI, D.—(1957) *Proc. nat. Acad. Sci., Wash.*, **43**, 857.
CLAUDE, A.—(1939) *Science*, **90**, 213.
COLTER, J. S. AND BROWN, R. A.—(1956) *Ibid.*, **124**, 1077.
COMMONER, B., LIPPINCOTT, J. A., SHEARER, GEORGIA, B., RICHMAN, ELLEN E. AND WU, JIA-HSI.—(1956) *Nature*, **178**, 767.
ELSON, D. AND CHARGAFF, E.—(1954) *Ibid.*, **173**, 1037.
EPSTEIN, M. A.—(1956) *Brit. J. Cancer*, **10**, 33.—(1957) *Ibid.*, **11**, 268.
FRAENKEL-CONRAT, H. AND WILLIAMS, R. C.—(1955) *Proc. nat. Acad. Sci., Wash.*, **41**, 690.
GAYLORD, W. H.—(1955) *Cancer Res.*, **15**, 80.
GEY, G. O. AND BANG, F. B.—(1951) *Trans. N.Y. Acad. Sci.*, **14**, 15.
*Iidem* AND GEY, M. K.—(1954) *Ann. N.Y. Acad. Sci.*, **58**, 976.
GIERER, A. AND SCHRAMM, G.—(1956) *Nature*, **177**, 702.
HERSHEY, A. D. AND CHASE, M. J.—(1952) *J. gen. Physiol.*, **36**, 39.
LIPPINCOTT, J. A. AND COMMONER, B.—(1956) *Biochim. biophys. Acta*, **19**, 198.
MOLONEY, J. B.—(1957) *J. nat. Cancer Inst.*, **18**, 515.
PARKER, R. C. AND RIVERS, T. M.—(1936) *J. exp. Med.*, **64**, 439.
ROUILLER, C., HAGUENAU, F., GOLDE, A. AND LACOUR, F.—(1956) *Bull. Ass. franç. Cancer*, **43**, 10.
SCHRAMM, C.—(1947) *Z. Naturforsch.*, 2*b*, 249.
UHLER, MARIANNE AND GARD, S.—(1954) *Nature*, **173**, 1041.